# An Account of the Practice of One of the Physicians of the Westminster General Dispensary, and of the Western Dispensary, from the 20th of December, 1806, to the 20th of January, 1807

**Published:** 1807-02

**Authors:** S. Fothergill

**Affiliations:** Southampton Street, Strand


*An Account of the Practice of one of the Physicians of the Westminster General Dispensary, and of the Western Dispensary, from the 20th of December,* 1806, *to the 20th of January,* 1807.

| | | | |
|---|---|---|---|
| ACUTE DISEASES. | | Pneumonia Notha | 4 |
| Catarrh | 5 | Asthenia | 8 |
| Typhus | 2 | Dyspepsia | 3 |
| Synochus | 3 | Gastrodynia | 5 |
| Acute Rheumatism | 4 | Diarrhœa | 3 |
| Enteritis | 1 | Dropsy | 2 |
| Measles | 3 | Chronic Rheumatism | 8 |
| Hooping Cough | 4 | Lumbago and Sciatica | 6 |
| Acute Diseases of Infants | 8 | Cephalalgia | 3 |
| CHRONIC DISEASES. | | Paralysis | 3 |
| Pulmonary Consumption | 3 | Cutaneous Diseases | 10 |
| Cough and Dyspnœa | 26 | Worms | 2 |
| Hæmoptoe | 3 | Menorrhagia | 3 |
| Asthma | 6 | Amenorrhœa | 5 |
| Pleurodyne | 5 | Inflamed Breast | 2 |

As usual at this season of the year, coughs and pulmonic affections are very prevalent. In my own practice, since the last Report, nothing particular has occurred.

In various parts of London, Westminster, the suburbs, and adjacent villages, the inhabitants have recently been considerably alarmed by the symptoms of canine madness attacking a great number of dogs; several men have been bitten, one manufacturer alone having sent six of his men to a surgeon to have the affected parts excised; and I have lately been informed that a poor wretch has miserably perished.

Under these circumstances, unless some active measures to destroy the affected animals are adopted, there being some probability of the disease becoming more general, I thought it might not be wholly unprofitable to give a short account of this terrible complaint, referring those who wish for further information on the subject, to the writings of Dioscorides, Galen, Cælius Aurelianus, Celsus, Ætius, Boerhaave, Mead, Fothergill, and Hamilton.

Dogs are most frequently affected with the disease in hot weather and in the extreme of cold; in the present season, which is temperate, canine madness has rapidly spread throughout most parts of Middlesex; proving, that when once the poison is produced, weather has little influence upon the infection. The first signs of the complaint in dogs, are an appearance of shyness and melancholy; avoiding their accustomed exercise, and shunning the notice and intercourse of men; general torpor and stupidity, a dull eye, deafness, and occasional growling: they now refuse their food, shewing great aversion to liquids; and the second stage may be said to begin, when they no longer know their masters, bieathe short, foam at the mouth, become emaciated, and as the disease advances evince the greatest horror of water, snap at every thing in their way, and run with fury as if blind, till they fall exhausted or are destroyed, seldom being suffered to die of the complaint; in their course their tail is kept close between their legs, their tongue hangs from the mouth, which distils foam; they bite at plants, animals, or whatever they meet, and if not killed very soon die, this furious state seldom lasting twenty-four hours. A strong decoction of the roots of the rosa sylvestris or rosa canina, was formerly thought very efficacious if given soon after the dog was observed to be affected.*

The symptoms of hydrophobia in man seldom appear immediately after the bite; sometimes several months elapse without any affection being observed. Boerhaave relates a case where twenty years had passed before the symptoms broke out, and another where they came on only one day after the poison was applied. The most usual time is from three to eight weeks, when the person who has had the misfortune to be bit begins to feel pain, similar to that of rheumatism, in the affected and contiguous parts; anxiety, inquietude, and uneasiness, with disturbed sleep and general lassitude succeed; the patient is thoughtful, much depressed in spirits, and seeks for solitude; respiration becomes painful, cold sweats bedew the surface of the body, which is frequently affected with spasmodic twitchings and general tremors; the pulse now and then intermits; all food, though swallowed, is disliked; and in the course of twenty-four hours these symptoms having having increased, the operation of swallowing is so difficult and painful that the patient has the utmost aversion for liquids, the very sight of which often affects him with horror, and produces a convulsive spasm in all the muscles subservient to deglutition. The voice is hoarse and sometimes resembles the growling of a dog; and from some noise being made in attempting to cough up the phlegm or saliva, a person is said to bark. Sleep cannot be obtained; there is often, though not always, a considerable degree of fever; priapismus urgens et seminis emissio frequens; the patient spits out the saliva with strong marks of disgust, being unable to swallow it; the convulsions become general, and delirium is frequently present, sometimes very violent, though much oftener the poor wretch is conscious of his state, is extremely depressed in mind, and warns the by-standers to be cautious lest he should hurt them; this deplorable degree of misery however seldom continues more than two days when death closes the tragic scene.

In the cure of hydrophobia we do not seem to be more successful than our predecessors; in the writings of the ancients many extraordinary cures are attested, but as similar means have since proved abortive, we are rather to hope for a preventive than a cure; of these many have been tried. Doctor Mead thought his famous powder, composed of ash-coloured ground liver wort (lichen cinereus terrestris) dried and powdered one drachm, and half a drachm of black pepper powdered, taken in half a pint of cow's milk warm, for four mornings successively, a most certain preventive; this, however, with another highly celebrated remedy, the Ormskirk medicine, having frequently failed, we are rather to suppose that in most cases said to be cured, the poison was never applied, and the patient consequently in no danger. Sea-bathing is equally uncertain. Boerhaave has recorded a case of a person ship-wrecked, who swam in the sea several hours; he had previously been bitten by a mad-dog, and the symptoms of hydrophobia appeared some days after he got on shore. Mercurial friction, so as to keep up a gentle salivation for fifteen or twenty days, has been recommended from high authority and large experience.* The only certain method however seems to be, that of *instantly* excising the affected part, cutting below the wound, and keeping up a discharge for some days. People are frequently said to have been smothered in the last stage of hydrophobia; this, however, is revolting to our feelings, and I believe is rather

* Sauvages' Nosology.

rather resorted to from the fear of the parties concerned than by the advice of the medical attendants; I should in such cases recommend the cold affusion, continued long and with some degree of violence; the shock to patients dreading the appearance of water is very great; and some instances are recorded, where, when the first surprise and consternation were ended, they recovered a calm and tranquil state, and the symptoms of the disease did not reappear.*

*Southampton Street, Strand,*
*January* 22, 1807.
S. FOTHERGILL.

